# Distribution and associated factors of psychological dimensions related to antimicrobial resistance countermeasures among hospital staff in Japan: an exploratory cross-sectional study

**DOI:** 10.1093/jacamr/dlag180

**Published:** 2026-08-21

**Authors:** Tatsuya Tai, Yuichi Muraki, Takahiro Motoki, Tatsuya Inoue, Masahiro Watanabe, Yusuke Fukumori, Taichi Ito, Yumiko Mashima, Kayo Fujimura, Kyoko Yokota, Hiroaki Tanaka, Shinji Kosaka, Yasuaki Mino, Teruki Dainichi

**Affiliations:** Department of Infection Control Service Office, Kagawa University Hospital, 1750-1 Ikenobe, Miki, Kagawa 761-0793, Japan; Department of Pharmacy, Kagawa University Hospital, 1750-1 Ikenobe, Miki, Kagawa, Japan; Department of Clinical Pharmacoepidemiology, Kyoto Pharmaceutical University, Kyoto, Japan; Department of Infection Control Service Office, Kagawa University Hospital, 1750-1 Ikenobe, Miki, Kagawa 761-0793, Japan; Department of Pharmacy, Kagawa University Hospital, 1750-1 Ikenobe, Miki, Kagawa, Japan; Department of Pharmacy, Kagawa University Hospital, 1750-1 Ikenobe, Miki, Kagawa, Japan; Department of Pharmacology, School of Pharmacy, Shujitsu University, 1-6-1 Nishigawara, Naka-ku, Okayama, Japan; Department of Infection Control Service Office, Kagawa University Hospital, 1750-1 Ikenobe, Miki, Kagawa 761-0793, Japan; Center of Infectious Disease Education, Kagawa University Hospital, 1750-1 Ikenobe, Miki, Kagawa, Japan; Department of Infection Control Service Office, Kagawa University Hospital, 1750-1 Ikenobe, Miki, Kagawa 761-0793, Japan; Center of Infectious Disease Education, Kagawa University Hospital, 1750-1 Ikenobe, Miki, Kagawa, Japan; Department of Infection Control Service Office, Kagawa University Hospital, 1750-1 Ikenobe, Miki, Kagawa 761-0793, Japan; Department of Nursing, Kagawa University Hospital, 1750-1 Ikenobe, Miki, Kagawa, Japan; Department of Infection Control Service Office, Kagawa University Hospital, 1750-1 Ikenobe, Miki, Kagawa 761-0793, Japan; Department of Nursing, Kagawa University Hospital, 1750-1 Ikenobe, Miki, Kagawa, Japan; Department of Infection Control Service Office, Kagawa University Hospital, 1750-1 Ikenobe, Miki, Kagawa 761-0793, Japan; Center of Infectious Disease Education, Kagawa University Hospital, 1750-1 Ikenobe, Miki, Kagawa, Japan; Department of Pharmacy, Kagawa University Hospital, 1750-1 Ikenobe, Miki, Kagawa, Japan; Department of Pharmacy, Kagawa University Hospital, 1750-1 Ikenobe, Miki, Kagawa, Japan; Department of Pharmacy, Kagawa University Hospital, 1750-1 Ikenobe, Miki, Kagawa, Japan; Department of Infection Control Service Office, Kagawa University Hospital, 1750-1 Ikenobe, Miki, Kagawa 761-0793, Japan; Dermatology, Kagawa University Hospital, 1750-1 Ikenobe, Miki, Japan

## Abstract

**Background:**

Research on antimicrobial resistance countermeasures primarily relies on the knowledge–attitude–practice (KAP) framework. However, knowledge gains do not reliably translate into behavioural change, and psychological dimensions beyond knowledge remain insufficiently characterized when considered concurrently within a single population.

**Objectives:**

To characterize four antimicrobial resistance-related psychological dimensions—risk perception, self-efficacy, sense of responsibility and attitude—among hospital staff and examine how they vary according to occupational and organizational factors.

**Methods:**

A cross-sectional survey was administered to the entire workforce at a Japanese university hospital in 2025. Psychological dimensions were assessed using a researcher-developed questionnaire. Risk perception was a three-item composite score (range: 3–15), and self-efficacy, sense of responsibility and attitude were each measured using single-item indicators. Multivariate linear regression with HC3 robust standard errors was used for the primary analysis, with occupational category, years of clinical experience, managerial position and infection control committee participation entered as covariates.

**Results:**

In total, 1077 staff members were analysed (response rate: 81.7%). Risk perception was positively associated with years of clinical experience, although this association was not consistent across models. Self-efficacy was lower among non-healthcare workers compared with nurses. Sense of responsibility was consistently lower among physicians, other healthcare workers and non-healthcare staff than in nurses. Attitudes showed no clear associations.

**Conclusions:**

This study extends previous KAP-based evidence by showing that associated factors vary across dimensions. Occupational differences in sense of responsibility emerged as the most consistent finding and may represent a dimension less amenable to knowledge-based interventions.

## Introduction

Antimicrobial resistance (AMR) is a global public health threat that may undermine modern medicine.^1,2^ Beyond its clinical toll, AMR imposes a substantial economic burden through prolonged hospitalization, increased costs of second-line treatment, and reduced productivity.^3–5^ In Japan, the First National Action Plan on AMR (2016–20, extended to 2022) placed the promotion of appropriate antimicrobial use and infection control in healthcare institutions at its centre.^6^ A Second Action Plan (2023–27) was subsequently formulated, maintaining the One Health structure while strengthening financial support for surveillance and stewardship.^7^ However, progress under the first plan was uneven across domains,^8,9^ and the implementation of national plans is frequently constrained by limited resources and personnel.^10,11^

The effectiveness of these measures ultimately depends on how staff behave in routine clinical practice. Within hospitals, antimicrobial stewardship programmes (ASPs) are central to optimizing antimicrobial use and limiting the emergence of AMR, and their clinical and economic benefits have been well established.^12^ In Japan, reimbursement incentives promote multidisciplinary ASP, engaging not only physicians but also non-prescribing occupations such as pharmacists, nurses and clinical laboratory technicians.^13^ Stewardship in practice therefore involves a broad range of staff beyond the dedicated team, including non-prescribing occupations whose psychological dimensions have rarely been examined. Accordingly, strengthening AMR countermeasures requires attention to the behaviours of this diverse workforce and to the psychology that underpins them.

Previous research on AMR countermeasures has extensively used the knowledge–attitude–practice (KAP) framework, and antimicrobial stewardship and infection control behaviours have been reported to vary substantially across institutions and occupational categories.^14,15^ Within Japan, differences in knowledge and attitudes across occupations and clinical departments have also been reported,^16,17^ and common infectious diseases continue to be treated with broad-spectrum antimicrobials.^18^ However, education-driven improvements in knowledge do not reliably translate into behavioural change,^19^ indicating that dimensions beyond knowledge must also be addressed.

Behavioural science research has shown, both theoretically and empirically, that multiple psychological dimensions—including risk perception,^20,21^ self-efficacy,^22^ sense of responsibility^23,24^ and attitude^25^—shape preventive and professional behaviours. However, in the context of AMR countermeasures, existing studies have remained concentrated within narrower frameworks, particularly KAP-based approaches.^14,15^ Moreover, the few studies that have examined dimensions beyond knowledge have typically addressed a single psychological construct in isolation.^21,26^ The psychology of non-prescribing, non-nursing occupations is rarely examined alongside that of physicians. Evidence that simultaneously captures multiple psychological dimensions in the same population and compares their dimension-specific associations with occupation, clinical experience, managerial status and organizational involvement remains limited.

In this study, we focused on four psychological dimensions considered directly relevant to AMR countermeasure practices—risk perception, self-efficacy, sense of responsibility and attitude—each grounded in an established behavioural science theory.^20–25^ These theories share the premise that behaviour is determined not by knowledge alone but also by appraisals such as how threatening a problem is perceived to be, whether one believes one can act, whether one regards the task as part of one's role and how one evaluates the countermeasures. We deliberately restricted our focus to intra-individual dimensions that are more directly modifiable through intervention; we did not measure other constructs within these theories, such as subjective norms, perceived behavioural control and outcome expectancies. Questionnaire items concerning the in-hospital environment and organization were addressed in secondary sensitivity analyses. Drawing on prior literature, we pre-specified directional hypotheses for how these psychological dimensions might relate to occupational and organizational factors: that a longer clinical experience would be associated with higher risk perception,^14,15^ that prior participation in an infection control committee would be associated with more favourable attitudes,^27^ that sense of responsibility would differ across occupational categories, given differences in professional roles within antimicrobial stewardship^28^ and that self-efficacy would similarly vary by occupational category, given differences in direct engagement with AMR countermeasures.^22^

We therefore aimed to characterize AMR-related psychological dimensions among hospital staff and to examine how these dimensions varied according to occupational category, years of clinical experience, managerial position and infection control committee participation. Characterizing these dimension-specific patterns may inform the design of more targeted, profession-specific approaches to strengthening AMR countermeasures.

## Methods

### Study design and participants

This cross-sectional study was conducted in 2025 among the entire workforce of Kagawa University Hospital (613 beds), a publicly owned national university teaching hospital in western Japan that serves as a tertiary referral centre for Kagawa Prefecture (population approximately 0.9 million); in Japan, public and private hospitals alike operate within a single universal social health insurance scheme with a nationally uniform fee schedule. The hospital operates a hospital-wide ASP led by an antimicrobial stewardship team (AST) comprising infectious disease physicians, dedicated pharmacists, nurses, and clinical laboratory technicians. Designated broad-spectrum and anti-methicillin-resistant Staphylococcus aureus (MRSA) agents (e.g. carbapenems and glycopeptides) require notification of use at initiation and when continued for 10 days or longer, and selected newer broad-spectrum agents (e.g. ceftazidime/avibactam and colistin) require prior AST approval at initiation (Table S1, available as Supplementary data at *JAC-AMR* Online). Pharmacists provide monitoring and feedback for anti-pseudomonal and anti-MRSA agents and perform therapeutic drug monitoring of vancomycin, teicoplanin and voriconazole. The hospital participates in national surveillance programmes (J-SIPHE and JANIS). Antimicrobials are prescribed by physicians and dentists, whereas pharmacists, nurses and clinical laboratory technicians support decision-making but do not prescribe (Table S2). Attendance at AMR-related training sessions twice per year is mandatory for all staff. In terms of stewardship structure and reimbursement-based AST operation, the hospital is considered broadly representative of Japanese university and tertiary-care hospitals, although it may differ from smaller community hospitals.

### Measures

To assess the psychological dimensions related to AMR countermeasures pragmatically, we developed an original questionnaire (Table S3). We did so because, to our knowledge, no validated scale simultaneously measures these four psychological dimensions in relation to AMR countermeasures; most existing AMR-related instruments are KAP-type knowledge and attitude checklists.^14,15^ Items were created from concepts and findings in the behavioural science and stewardship literature—the Health Belief Model,^20,21^ Social Cognitive Theory,^22^ the Theory of Planned Behaviour^25^ and studies of professional roles and responsibilities in infection prevention and stewardship^23,24^—and each item was pre-assigned to one of the four dimensions. Whereas the theoretical structure of the items drew on these behavioural science theories, the practical, context-specific item wording was adapted from Sneddon *et al*.,^19^ a study evaluating the effect of stewardship training in two hospitals. That study assessed pre- and post-training changes in knowledge and attitudes and was not validated as a multidimensional psychological scale; accordingly, we referred to it as a source of concrete, field-tested item wording rather than as a validated KAP scale.

No formal pilot study of the questionnaire was conducted; however, before administration, members of the hospital's AST and infection control team reviewed the draft and confirmed its content validity. The absence of formal piloting and validation is acknowledged as a limitation.

All items were rated on a five-point Likert scale (1 = strongly disagree, 2 = disagree, 3 = neither agree nor disagree, 4 = agree, 5 = strongly agree). In addition to items reflecting individual psychological dimensions, the questionnaire included items addressing in-hospital environment and organizational factors. For this study, six items considered to reflect individual psychological dimensions (Q1–5 and Q8) were included in the analysis, and two items addressing environmental and organizational factors (Q6 and Q7) were excluded from the primary analysis. The six items were pre-assigned to four psychological dimensions based on prior literature and conceptual alignment: risk perception,^20,21^ self-efficacy,^22^ sense of responsibility^23,24^ and attitude.^25^

Items Q1–3 were conceptualized as multiple indicators of risk perception. Prior to calculating the composite score, internal consistency was acceptable (Cronbach’s α = 0.87), exceeding the conventional threshold of 0.70;^29^ the sum of the three items was then used as the risk perception score (range 3–15). Q4 (attitude), Q5 (self-efficacy) and Q8 (sense of responsibility) were treated as single-item indicators. The full questionnaire administered to respondents is presented in the original Japanese together with an English translation (Table S3).

Occupational categories [nurse (reference), physician, pharmacist, other healthcare worker, non-healthcare worker], years of clinical experience (continuous variable),^14,15^ managerial position (leadership role or general staff)^27^ and infection control committee participation (presence or absence within the preceding 5 years)^27^ were collected as background characteristics.

Occupational categories and managerial positions were operationally defined as follows: Other healthcare workers included radiologic technologists, physiotherapists, occupational therapists, speech-language-hearing therapists, clinical laboratory technicians, clinical engineers, orthoptists, registered dietitians, dental hygienists, medical social workers, in-hospital clinical research coordinators and nursing assistants. Non-healthcare workers included administrative staff, outpatient clerks, medical clerks, medical record managers, community liaison staff, contracted staff, system administrators and medical equipment operators. Managerial positions comprised professors, associate professors, senior lecturers, assistant professors, head nurses, deputy head nurses, deputy directors of nursing and department directors. These data were not collected via a questionnaire; instead, they were obtained by linking the participant data to institutional human resources records using staff identification numbers. Following data linkage, research identification numbers were assigned and all personal identifiers, including names and staff identification numbers, were removed prior to analysis.

### Statistical analysis

Descriptive statistics were summarized for all questionnaire items. For the three risk perception items (Q1–3), internal consistency was confirmed using Cronbach’s α prior to calculating the composite score.^29^ Floor and ceiling effects were defined as more than 15% of the responses occurring at the minimum and maximum values, respectively.^30^ The distribution of scores for each psychological dimension was displayed using histograms and bar charts.

Multivariate linear regression was performed, with each psychological dimension score modelled separately as the dependent variable. The independent variables included occupational category [nurses (reference), physicians, pharmacists, other healthcare workers, and non-healthcare workers], years of clinical experience (continuous), managerial position (leadership role or general staff) and infection control committee participation (within the preceding 5 years). The effect of each independent variable was expressed as a regression coefficient (β) with 95% confidence intervals (95% CIs).

Linear regression treating Likert scale responses as continuous variables has been employed in prior research and was adopted as the primary analysis in this study.^31^ However, given that Q4, Q5 and Q8 were single-item ordinal variables and the risk perception score is a sum score ranging from 3 to 15, none fully satisfied the assumption of continuous measurement. To address the potential for skewed distributions and heteroscedasticity, HC3 robust standard errors—a conservative approach for small-to-moderate samples—were applied.^32^ To further assess the robustness of the results, ordinal logistic regression was conducted as a sensitivity analysis and directional consistency with the primary analysis was used as evidence of robustness.

Model assumptions were examined using residual and normal probability plots. Multicollinearity was assessed using variance inflation factor (VIF). The overall model fit was evaluated using F-statistic and *R*^2^ values. Separate regression analyses were conducted for each predefined indicator of the psychological dimensions (risk perception score, Q4, Q5 and Q8) modelled as the dependent variables. Multiple dimension scores were not entered simultaneously into a single model because of conceptual overlap and the anticipated high intercorrelations.

As this study was designed to generate hypotheses, the direction and consistency of the associations were prioritized over explanatory power. Interpretation was based primarily on β coefficients and 95% CIs. An association was described as showing a ‘directional trend’ when the 95% CI did not include zero, and as showing ‘no clear direction’ when it did. *P* values were reported as supplementary reference information only; binary judgements based on statistical significance were not made, and no correction for multiple comparisons was applied.

Four sensitivity analyses were pre-specified. First, given that the managerial position variable collapsed academically ranked physicians (professor, associate professor, senior lecturer and assistant professor), nursing leaders (head nurse, deputy head nurse and deputy director of nursing) and department directors into a single binary variable despite substantial heterogeneity in AMR-related roles across these positions, a model excluding managerial positions was conducted to assess the potential influence of this heterogeneity on the estimates. Second, ordinal logistic regression was performed with each dependent variable treated as ordinal. Third, a score summing the two environmental/organizational items (Q6 and Q7) that had been excluded from the primary analysis was added as a covariate to each model. Fourth, sex (binary: female/male) was added as a covariate to each model. For each analysis, directional consistency with the primary analysis was used to evaluate robustness. These sensitivity analyses were conducted to assess the robustness of the primary analysis findings and were not intended to identify new associations. Because subgroups such as pharmacists and infection control committee participants were small and no correction for multiple comparisons was applied in this exploratory design, associations observed only in the sensitivity analyses were not emphasized and were treated as hypotheses requiring confirmation. All analyses were performed using R version 4.4.3 (R Foundation for Statistical Computing, Vienna, Austria).

### Ethics statements

This study involved the secondary use of data from an anonymous survey administered online to the entire workforce as part of routine hospital operations. The primary purpose of the survey was self-assessment within training, and participation was embedded in the training framework. Information on occupational category, years of clinical experience, managerial position and infection control committee participation was obtained by linking participant data to institutional human resources records using staff identification numbers. After data linkage, personal identifiers, including names and staff identification numbers, were removed by the research team before analysis. No new surveys or research interventions were conducted. Accordingly, this study was exempted from ethical review by the Ethics Committee of Kagawa University (approval number: 2025-205).

## Results

### Participants

In 2025, a survey assessing psychological dimensions related to AMR countermeasures was administered to 1508 hospital staff members, of whom 1232 responded (response rate: 81.7%). Of these, 11 respondents with unidentifiable staff identification numbers and 144 with implausible values for years of clinical experience (124 years, *n* = 138; 125 years, *n* = 6) were excluded. The observed range of clinical experience in the analysed sample was 0–35 years; values of 124 and 125 years deviated markedly from this range and were considered likely default values generated by the institutional human resources system when the date of hire could not be calculated. The final analytic sample included 1077 participants (exclusion rate, 12.6% of respondents) (Table S4).

Participant characteristics are presented in Table 1. The median clinical experience was 10.0 years [interquartile range (IQR): 6.0–16.0]. The sample included 529 nurses (49.1%), 212 physicians (19.7%), 41 pharmacists (3.8%), 140 other healthcare workers (13.0%) and 155 non-healthcare workers (14.4%). Overall, 194 participants (18.0%) held managerial positions, and 40 (3.7%) had participated in an infection control committee within the preceding 5 years. Overall, 767 participants (71.2%) were female and 310 (28.8%) were male.

**Table 1.** Participant characteristics (*n* = 1077)

Factor	Overall
Total participants	1077
Years of clinical experience	10.0 (6.0–16.0)
<5 years	160 (14.9)
5–9 years	374 (34.7)
10–19 years	401 (37.2)
≥20 years	142 (13.2)
Occupational category	
Nurse	529 (49.1)
Physician	212 (19.7)
Pharmacist	41 (3.8)
Other healthcare worker	140 (13.0)
Non-healthcare worker	155 (14.4)
Managerial position (leadership role)	194 (18.0)
Infection control committee participation (within the preceding 5 years)	40 (3.7)

Continuous variables are presented as medians (IQR), and categorical variables are presented as numbers (%).

### Descriptive statistics of AMR-related psychological dimensions

**Figure 1.**
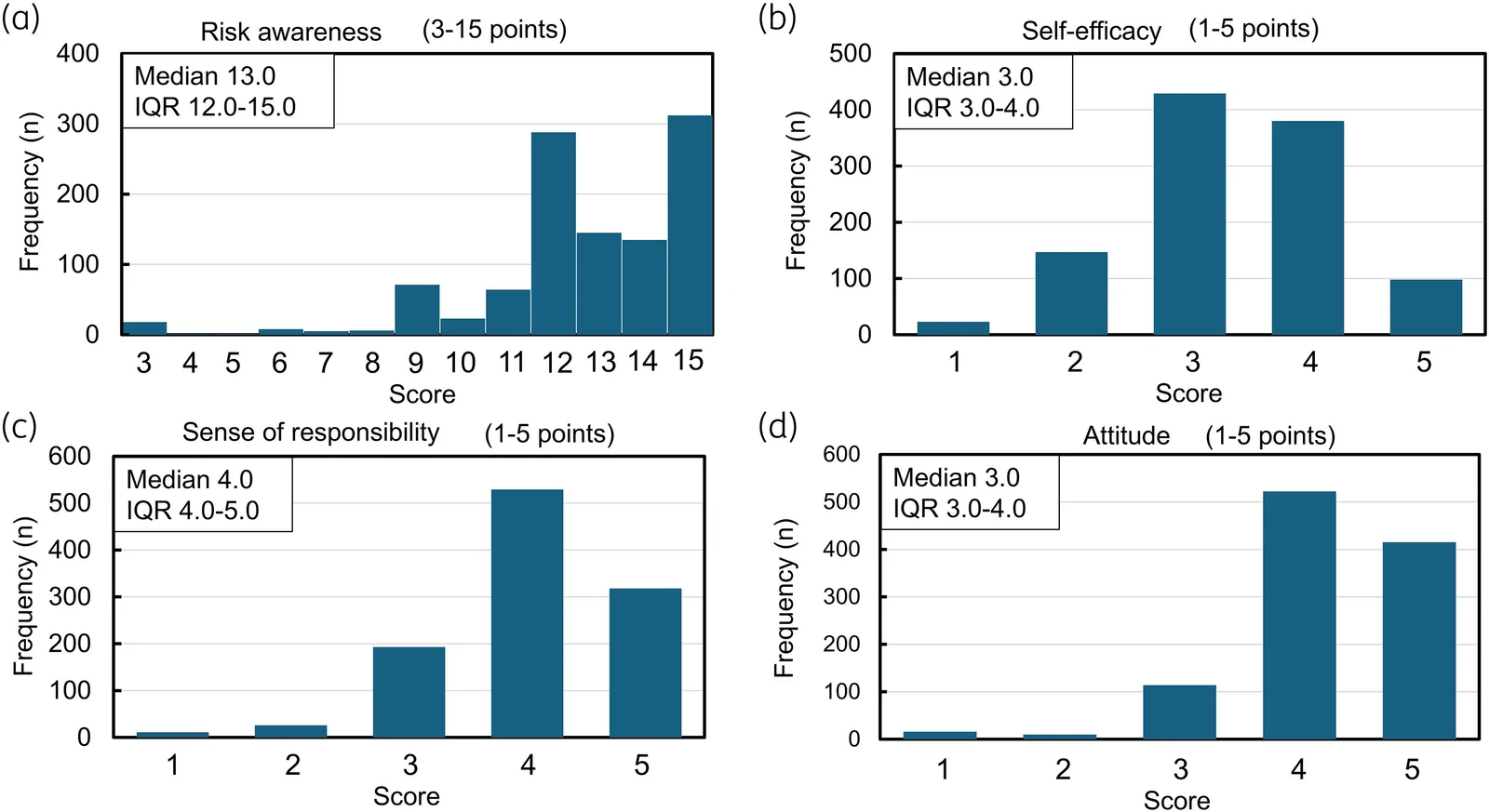
Frequency distributions of AMR-related psychological dimension scores among hospital staff (*n* = 1077). (a) Distribution of the risk perception score (range 3–15), calculated as the sum of three items (Q1–Q3); internal consistency was Cronbach's α = 0.87. Distributions of (b) Q5 (self-efficacy), (c) Q8 (sense of responsibility) and (d) Q4 (attitude), respectively. Each is a single-item indicator rated on a five-point Likert scale (range 1–5). Median (IQR) values for each dimension were as follows: risk perception 13.0 (12.0–15.0), self-efficacy 3.0 (3.0–4.0), sense of responsibility 4.0 (4.0–5.0) and attitude 3.0 (3.0–4.0).

The distribution of scores for each psychological dimension is shown in Figure 1. A ceiling effect was observed for the risk perception score, with 312 participants (29.0%) achieving maximum value of 15 (Figure 1a). No floor or ceiling effects were observed for the remaining dimensions.

The median (IQR) scores for each dimension were presented in Table 2: risk perception 13.0 (12.0–15.0), self-efficacy 3.0 (3.0–4.0), sense of responsibility 4.0 (4.0–5.0) and attitude 3.0 (3.0–4.0).

**Table 2.** Descriptive statistics of AMR-related psychological dimension scores (*n* = 1077)

Score	Median (IQR)
Risk perception	13.0 (12.0–15.0)
Self-efficacy	3.0 (3.0–4.0)
Sense of responsibility	4.0 (4.0–5.0)
Attitude	3.0 (3.0–4.0)

Score distributions are presented as medians (IQR). The risk perception score was calculated as the sum of multiple pre-assigned items (range: 3–15). Self-efficacy, sense of responsibility and attitude were calculated as single-item indicators (range 1–5 each).

### Factors associated with AMR-related psychological dimension scores

The results of the multivariate linear regression analysis are presented in Table 3. The sample size for all regression models was 1077. Model assumptions were assessed using residual and normal probability plots, and no serious violations were identified. Multicollinearity was assessed using VIF. All independent variables had a VIF <2 (maximum: 1.53), indicating no problematic multicollinearity.

**Table 3.** Factors associated with AMR-related psychological dimension scores

Independent variable	Risk perception β (95% CI), *P*	Self-efficacy β (95% CI), *P*	Sense of responsibility β (95% CI), *P*	Attitude β (95% CI), *P*
Physician (versus nurse)	−0.254 (−0.677, 0.170), *P* = 0.240	0.137 (−0.007, 0.280), *P* = 0.063	−0.225 (−0.354, −0.095), *P* < 0.001	−0.078 (−0.224, 0.067), *P* = 0.292
Pharmacist (versus nurse)	0.722 (0.154, 1.290), *P* = 0.013	0.123 (−0.125, 0.370), *P* = 0.331	−0.019 (−0.262, 0.224), *P* = 0.879	0.200 (−0.011, 0.411), *P* = 0.063
Other healthcare worker (versus nurse)	−0.213 (−0.669, 0.243), *P* = 0.360	0.060 (−0.097, 0.216), *P* = 0.453	−0.309 (−0.466, −0.153), *P* < 0.001	−0.081 (−0.232, 0.070), *P* = 0.292
Non-healthcare worker (versus nurse)	0.004 (−0.407, 0.415), *P* = 0.986	−0.153 (−0.295, −0.010), *P* = 0.036	−0.789 (−0.940, −0.637), *P* < 0.001	−0.046 (−0.186, 0.093), *P* = 0.515
Years of clinical experience	0.024 (0.004, 0.045), *P* = 0.022	0.003 (−0.004, 0.010), *P* = 0.341	0.002 (−0.005, 0.009), *P* = 0.551	0.006 (−0.001, 0.013), *P* = 0.117
Managerial position (leadership role)	0.182 (−0.263, 0.627), *P* = 0.423	0.060 (−0.086, 0.205), *P* = 0.420	0.071 (−0.066, 0.208), *P* = 0.309	0.081 (−0.072, 0.234), *P* = 0.297
Infection control committee participation	0.020 (−1.014, 1.053), *P* = 0.970	0.088 (−0.208, 0.385), *P* = 0.560	0.198 (−0.081, 0.477), *P* = 0.164	0.149 (−0.164, 0.462), *P* = 0.351
*n*	1077	1077	1077	1077

Values represent regression coefficients (β), 95% CIs and *P* values. Nurses served as the reference category for occupational categories.

For risk perception, a positive directional trend was observed for pharmacists (β = 0.722, 95% CI: 0.154, 1.290, *P* = 0.013) and years of clinical experience (β = 0.024, 95% CI: 0.004, 0.045, *P* = 0.022). No clear directional trends were observed for any other independent variables.

For self-efficacy, a negative directional trend was observed for non-healthcare workers (β = −0.153, 95% CI: −0.295, −0.010, *P* = 0.036). No clear directional trends were observed for any other independent variables.

For sense of responsibility, negative directional trends were observed for physicians, other healthcare workers, and non-healthcare workers compared with nurses (physician: β = −0.225, 95% CI: −0.354, −0.095, *P* < 0.001; other healthcare worker: β = −0.309, 95% CI: −0.466, −0.153, *P* < 0.001; non-healthcare worker: β = −0.789, 95% CI: −0.940, −0.637, *P* < 0.001).

Regarding attitudes, no clear directional trend was observed for any independent variable (Table 3).

### Sensitivity analyses

Results of the sensitivity analyses were presented in Tables S5–S7 and S9.

In the sensitivity analysis excluding managerial position, occupational differences in sense of responsibility were maintained in directions and magnitudes broadly consistent with the primary analysis (physician: β = −0.195, 95% CI: −0.319, −0.072, *P* = 0.002; other healthcare worker: β = −0.318, 95% CI: −0.473, −0.163, *P* < 0.001; non-healthcare worker: β = −0.793, 95% CI: −0.944, −0.642, *P* < 0.001). The directions and magnitudes of β coefficients for other independent variables were also broadly consistent with the primary analysis, with no major changes in the main patterns (Table S5).

In the sensitivity analysis using ordinal logistic regression, results were expressed as odds ratios (ORs) with 95% CIs. Occupational differences in the sense of responsibility—with lower scores among physicians, other healthcare workers and non-healthcare workers—and the negative trend in self-efficacy for non-healthcare workers were directionally consistent with the primary analysis. The positive directional trend between risk perception and years of clinical experience was also maintained, although the lower bound of the CI only marginally exceeded 1 (OR = 1.021, 95% CI: 1.004, 1.037, *P* = 0.013). For risk perception among pharmacists, a positive directional trend was observed in the primary analysis, but the CI crossed 1 in the ordinal logistic regression (OR = 1.606, 95% CI: 0.908, 2.841, *P* = 0.105), indicating limited cross-model consistency. No clear directional trend was observed for non-healthcare workers for risk perception in either model. Regarding attitude, infection control committee participation showed no clear directional trend in the primary analysis but showed a positive trend in the ordinal logistic regression (OR = 2.065, 95% CI: 1.063, 4.011, *P* = 0.032) (Table S6).

In the sensitivity analysis with the environmental score added as a covariate, this score was strongly and positively associated with self-efficacy (β = 0.304, 95% CI: 0.273, 0.335, *P* < 0.001). The lower self-efficacy among non-healthcare workers observed in the primary analysis (β = −0.153, *P* = 0.036) was largely attenuated (β = 0.030, *P* = 0.605). By contrast, the occupational differences in sense of responsibility (lower scores among physicians, other healthcare workers, and non-healthcare workers) remained consistent after adjustment for the environmental score (all *P* < 0.001). The environmental score was positively associated with all four dimensions (all *P* < 0.001) (Table S7).

The distribution of sex differed markedly across occupational categories (Table S8: 89.8% of nurses, 29.7% of physicians and 84.5% of non-healthcare workers were female). When sex was added as a covariate (Table S9, HC3 robust standard errors), the occupational differences in sense of responsibility were maintained, remaining clear for other healthcare workers (β = −0.256, 95% CI: −0.419, −0.093, *P* = 0.002) and non-healthcare workers (β = −0.752, 95% CI: −0.907, −0.598, *P* < 0.001) and, though attenuated, for physicians (β = −0.152, 95% CI: −0.301, −0.004, *P* = 0.044). Notably, non-healthcare workers showed the largest reduction in sense of responsibility despite being predominantly female, which is opposite to the direction that a simple sex effect would predict. The lower self-efficacy among non-healthcare workers was also maintained after adjustment for sex (β = −0.160, *P* = 0.027). Male sex was associated with lower risk perception (β = −0.447, *P* = 0.022) but was not significant for the other dimensions. Multicollinearity was again confirmed in the model including sex, with all VIFs below 2 (maximum 1.808 for physician occupation).

## Discussion

In this single-centre cross-sectional study, we characterized AMR-related psychological dimensions among hospital staff. Although prior AMR research has largely relied on specific frameworks, such as the KAP framework, in which knowledge has been the dominant focus,^14,15^ knowledge alone does not reliably translate into behavioural change.^19^ The present study revealed that four dimensions—risk perception, self-efficacy, sense of responsibility and attitude—follow distinct patterns of association with occupational and organizational factors, suggesting that the psychological foundations of AMR-related practice are not uniform and may require dimension-specific approaches. A ceiling effect was observed for risk perception scores, and occupational differences in sense of responsibility emerged as the most consistent signal. The negative trend in self-efficacy among non-healthcare workers was directionally consistent across the sensitivity analyses. By contrast, the association between risk perception and years of clinical experience showed limited cross-model consistency, and attitude showed no clear association with any examined factor.

Regarding risk perception, concentration at the maximum value was observed. Possible explanations for this ceiling effect include the impact of AMR education, item wording, and social desirability bias, although the present study provides no basis for prioritizing any single interpretation. Regardless of the underlying cause, the ceiling effect likely limits detection of individual variations and reduces statistical sensitivity. The distribution of sense of responsibility scores was skewed towards higher values, which may reflect the internalization of infection control as part of occupational role identity among healthcare professionals^23,24^; however, social desirability bias cannot be excluded. The self-efficacy and attitude scores were distributed around the median, with residual variation among staff members. These dimensions are strongly shaped by practical experience and the work environment,^22,25^ suggesting that they may be less amenable to improvement through educational interventions alone.

The positive directional trend between risk perception and years of clinical experience in the primary analysis aligned with the pre-specified hypothesis.^20,21^ However, in the ordinal logistic regression, the lower bound of the CI only marginally exceeded 1.000, and the association was not robust across models. Given the marked ceiling effect in risk perception score distribution, the possibility that restricted score variability may have distorted. Considering the results of both models together, the association between risk perception and years of clinical experience remains uncertain and should be cautiously interpreted. The positive directional trend observed for pharmacists was not pre-specified and may represent a chance finding. Given the small sample size, no substantive conclusion can be drawn without confirmation in a larger sample.

Regarding self-efficacy, a negative directional trend was observed among non-healthcare workers. Although occupational differences in self-efficacy were pre-specified, the only occupational category showing a clear directional pattern was non-healthcare workers.^22^ Self-efficacy is shaped not only by individual perceptions of capability but also through interaction with available resources and support structures in the environment.^22^ Among non-healthcare workers with little to no direct involvement in AMR countermeasures, limited opportunities to engage in these practices may partly explain lower self-efficacy. Indeed, in the sensitivity analysis with the environmental score added, this association was largely attenuated, which is consistent with an environmental contribution; however, given the conceptual proximity between these self-reported items, it should be interpreted as a correlational finding. In the other sensitivity analyses, the direction was maintained relative to the primary analysis. A positive directional trend was observed for physicians, but the CI crossed zero, precluding substantive interpretation.

Regarding sense of responsibility, negative directional trends were observed for physicians, other healthcare workers and non-healthcare workers compared with nurses, consistent with the pre-specified hypothesis that nurses would demonstrate a higher sense of responsibility than other occupational categories.^28^ This finding was largely preserved across fourth sensitivity analyses and is therefore the most robust exploratory finding of the present study. Prior research has reported that occupational categories differ in their involvement in AMR countermeasures and in their opportunities to practice infection control in daily work. The occupational differences observed here are directionally consistent with these prior findings.^14–17^ Nevertheless, the present study design cannot identify the underlying mechanisms.

Regarding attitudes, no clear directional association was observed with any independent variable in the primary analysis. Of note, infection control committee participation, which had been pre-specified as a potential correlate, demonstrated a positive directional trend in the ordinal logistic regression, although the small sample size precludes firm interpretation. Attitude is a multidimensional construct, and measurements with a single-item indicator may have been insufficient to detect associations with occupational category or years of clinical experience.^25^

From a practical standpoint, because the dimensions differ in how readily they can be changed, uniform education alone is likely insufficient. In particular, occupational differences in sense of responsibility may be better addressed through role design—such as embedding stewardship responsibilities into workflows and formalizing the involvement of non-prescribing occupations—than through attempts to change attitudes. Accessible guidance, decision support, and feedback may be effective for self-efficacy, whereas the ceiling effect for risk perception suggests limited scope for further awareness-raising. Locating these dimensions within a framework that distinguishes capability, opportunity and motivation, such as capability, opportunity, motivation, and behavior, may help design interventions tailored to specific dimensions and occupations.

This study had some limitations. First, these interpretations need to be situated within the structural, economic, and social factors that shape antimicrobial use beyond individual psychology, which a single-centre, self-reported design cannot adequately capture.^33^ Second, because of cross-sectional design, no causal inferences can be drawn between AMR-related psychological dimensions and behaviour. Third, the questionnaire was developed specifically for this study; in particular, self-efficacy, attitude, and sense of responsibility were assessed using single-item indicators, limiting structural validity and reliability. Furthermore, the measurement precision differed between the composite risk perception score and the single-item indicators, which should be considered when interpreting cross-dimensional differences. Fourth, because this was a single-centre study, the generalizability of the findings may be limited. Fifth, the frequency and content of educational exposure were not quantified and unmeasured confounding may remain. Sixth, the small sample size of pharmacists and infection control committee participants introduced uncertainty into these estimates. Seventh, because the data were self-reported, social desirability bias may have influenced responses. Eighth, this study was a secondary use of an anonymous survey, and because background variables were obtained by linking only respondents to institutional human resources records, background data for the entire invited population, including non-respondents, were unavailable; responder bias therefore cannot be excluded, as respondents and non-respondents could not be compared. Ninth, the participants excluded due to implausible values for years of clinical experience were disproportionately nurses, which may have affected the representativeness of the nurse group as the reference category and introduced bias into the occupational comparisons.

### Conclusions

This study characterized AMR-related psychological dimensions among hospital staff and examined their associations with occupational and organizational factors. Studies that simultaneously examine multiple psychological dimensions in the context of AMR countermeasures within a single population remain scarce; this study provides preliminary evidence addressing that gap. Previous KAP-based studies have primarily documented occupational differences in knowledge; the present study extends this evidence by demonstrating that associated factors vary across psychological dimensions, with the most robust signal observed for sense of responsibility. Occupational differences in sense of responsibility emerged as the most consistent finding, suggesting that this dimension may not be fully addressed by knowledge-based approaches alone and supporting hypotheses for profession-specific interventions. As all findings are exploratory, they should be treated as hypotheses requiring confirmation. Future studies should develop and validate multi-item scales and use longitudinal designs to evaluate the relationship between psychological dimensions and behaviour over time.

## Supplementary Material

dlag180_Supplementary_Data

## Data Availability

Data are available upon request and subject to privacy, ethics, and other restrictions.

## Transparency declarations

Y.M. received grants from Pfizer Japan Inc., Kowa Company Ltd., the Japan Pharmaceutical Association, the Japan Society for the Promotion of Science, and the Ministry of Health, Labor and Welfare. However, this study was not directly funded. Y.M. is a board member of the Japanese Society for Infection Prevention and Control and a committee member of the AMR Clinical Reference Center. Y.M. is editor-in-chief of the *Journal of Pharmaceutical Health Care and Sciences*. Y.M. purchased or provided data from DeSC Healthcare, Inc.; JMDC Inc.; Medical Data Vision Co., Ltd.; JAPIC; and H&H CONNECT Co., Ltd. at academic discount prices or equivalent conditions. These companies had no role in the study design, data analysis and interpretation, or writing of the manuscript. The remaining authors have no conflicts of interest to declare.

## Supplementary data

Tables S1–S8 is available as Supplementary data at *JAC-AMR* Online.
