# Supplementary material for: Distribution and associated factors of psychological dimensions related to antimicrobial resistance countermeasures among hospital staff in Japan: an exploratory cross-sectional study
Source: JAC Antimicrob Resist. 2026 Aug 21;8(4):dlag180. doi: 10.1093/jacamr/dlag180 (PMC13494903; doi:10.1093/jacamr/dlag180)
Supplement: Supplementary file 1 [file dlag180_supplementary_data.docx]

**Table S1. Antimicrobials subject to AST notification of use and prior approval**

Notification of use is required for the following antimicrobials.

| **Antimicrobial (generic name)** | **Notification form** | **Timing** |
| --- | --- | --- |
| Linezolid; vancomycin; teicoplanin; daptomycin | Anti-MRSA agent notification | At initiation and when continued for ≥10 days |
| Meropenem; doripenem | Broad-spectrum antimicrobial (carbapenem) notification | At initiation and when continued for ≥10 days |
| Levofloxacin | Broad-spectrum antimicrobial (non-carbapenem, excluding PIPC/TAZ) notification | At initiation and when continued for ≥10 days |

Prior AST approval is required for the following antimicrobials.

| **Antimicrobial (generic name)** | **Timing** |
| --- | --- |
| Ceftolozane/tazobactam; colistin; ceftazidime/avibactam; tigecycline; imipenem/cilastatin/relebactam; arbekacin; imipenem/cilastatin; ciprofloxacin; tedizolid | At initiation |

**Table S2. Representative AMR countermeasures by occupational category**

| **Occupational category** | **Representative practices** |
| --- | --- |
| Physician | Guideline-concordant empirical and definitive prescribing; obtaining cultures before starting antimicrobials; de-escalation; intravenous-to-oral switch |
| Pharmacist | Prospective audit and feedback; therapeutic drug monitoring; dose optimisation; management of restricted and formulary agents |
| Nurse | Timely and accurate specimen collection; accurate administration and timing; prevention of device-related infections |
| Other healthcare worker (e.g., clinical laboratory technician) | Rapid diagnostics; susceptibility testing and antibiogram reporting; timely communication of critical values |
| Non-healthcare worker | Limited direct clinical role |

Table S3. Questionnaire items for assessing AMR-related psychological dimensions among hospital staff

| Item | Psychological dimension |
| --- | --- |
| Q1: I believe antimicrobial resistance is a serious problem in clinical settings. | Risk perception |
| Q2: I feel anxious that antimicrobials may become ineffective due to antimicrobial-resistant bacteria. | Risk perception |
| Q3: I am concerned that patients in our hospital may become infected with antimicrobial-resistant bacteria. | Risk perception |
| Q4: I believe that following nationally and regionally recommended guidelines for antimicrobial use and infection control helps prevent the spread of antimicrobial resistance. | Attitude |
| Q5: I am confident that I can appropriately carry out AMR-related practices within my own work. | Self-efficacy |
| Q6: I can easily access infection control manuals and antimicrobial-related procedures in our hospital. | Environmental factor ※ |
| Q7: I find it easy to implement infection control and antimicrobial-related hospital rules in my daily work. | Environmental factor ※ |
| Q8: I believe that engaging in AMR countermeasures is part of my professional role. | Sense of responsibility |

Q6 and Q7 address environmental and organisational factors rather than individual psychological dimensions and were therefore excluded from the analysis.

**抗菌薬スチュワードシップ行動調査**

**抗菌薬耐性および抗菌薬スチュワードシップに関するご意見**

以下の記述にどの程度同意するか、それぞれの記述に1つずつチェックを入れてください。

|  |  | 強く反対 | 反対 | 賛成でも反対でもない | 同意 | 強く同意 |
| --- | --- | --- | --- | --- | --- | --- |
|  |  | 1 | 2 | 3 | 4 | 5 |
| 1 | 薬剤耐性は医療現場にとって深刻な問題であると思う。 |  |  |  |  |  |
| 2 | 薬剤耐性のある細菌によって、抗菌薬が効かなくなることに不安を感じている。 |  |  |  |  |  |
| 3 | 院内で薬剤耐性菌に感染する人が出ることを心配している。 |  |  |  |  |  |
| 4 | 国や地域で推奨されている抗菌薬の使い方や感染対策の指針に従うことは、薬剤耐性の拡大防止に役立つと思う。 |  |  |  |  |  |
| 5 | 自分の業務の中で、薬剤耐性に関係する対応を適切に行う自信がある。 |  |  |  |  |  |
| 6 | 院内の感染対策マニュアルや、抗菌薬に関する手順書には簡単にアクセスできる |  |  |  |  |  |
| 7 | 感染対策や抗菌薬に関する院内ルールは、日常業務の中で実践しやすいと感じる。 |  |  |  |  |  |
| 8 | 薬剤耐性対策に取り組むことは、私の専門職としての役割の一部だと思う。 |  |  |  |  |  |

ご意見ありがとうございました。

※参考文献：Jacqueline Sneddon, Lesley Cooper, Daniel Kwame Afriyie, Israel A Sefah, Alison Cockburn, Frances Kerr, Elaine Cameron, Joanna Goldthorpe, Amanj Kurdi, R Andrew Seaton, Supporting antimicrobial stewardship in Ghana: evaluation of the impact of training on knowledge and attitudes of healthcare professionals in two hospitals, JAC-Antimicrobial Resistance, Volume 2, Issue 4, December 2020, dlaa092, https://doi.org/10.1093/jacamr/dlaa092

Table S4. Occupational distribution of analysed and excluded participants

|  | Analysed participants | Excluded (implausible experience values) | Excluded (unidentifiable ID) |
| --- | --- | --- | --- |
| Occupational category, n (%) |  |  |  |
| Nurse | 529 (49.1) | 84 (58.3) | 0 (0) |
| Physician | 212 (19.7) | 24 (16.7) | 1 (9.1) |
| Pharmacist | 41 (3.8) | 4 (2.8) | 0 (0) |
| Other healthcare worker | 140 (13.0) | 13 (9.0) | 0 (0) |
| Non-healthcare worker | 155 (14.4) | 19 (13.2) | 9 (81.8) |
| Unknown | ― | 0 (0) | 1 (9.1) |
| Total | 1077 (100) | 144 (100) | 11 (100) |
| Psychological dimension scores, median [IQR] |  |  |  |
| Risk perception (range 3–15) | 13.0 [12.0–15.0] | 13.0 [13.0–15.0] | 11.0 [11.0–12.0] |
| Self-efficacy (range 1–5) | 3.0 [3.0–4.0] | 3.0 [3.0–4.0] | 3.0 [3.0–3.0] |
| Sense of responsibility (range 1–5) | 4.0 [4.0–5.0] | 5.0 [4.0–5.0] | 3.0 [3.0–5.0] |
| Attitude (range 1–5) | 3.0 [3.0–4.0] | 4.0 [4.0–5.0] | 4.0 [3.0–4.0] |

The occupational distribution (number and percentage) of the analysed participants (n = 1,077) and excluded participants (n = 155) is presented. The excluded participants were those with unidentifiable staff identification numbers (n = 11) and those with implausible values for years of clinical experience (124 or 125 years) whose records were deemed unreliable (n = 144). Occupations were classified into five groups: nurses, physicians, pharmacists, other healthcare workers, and non-healthcare workers.

Table S5. Sensitivity analysis excluding managerial position (n = 1077)

| Independent variable | Risk perception β (95% CI), p | Self-efficacy β (95% CI), p | Sense of responsibility β (95% CI), p | Attitude β (95% CI), p |
| --- | --- | --- | --- | --- |
| Physician  (vs nurse) | -0.179 (-0.556, 0.198), p=0.352 | 0.161 (0.033, 0.289), p=0.014 | -0.195 (-0.319, -0.072), p=0.002 | -0.045 (-0.175, 0.086), p=0.502 |
| Pharmacist  (vs nurse) | 0.695 (0.130, 1.259), p=0.016 | 0.114 (-0.133, 0.360), p=0.366 | -0.029 (-0.271, 0.212), p=0.811 | 0.188 (-0.022, 0.398), p=0.080 |
| Other healthcare worker (vs nurse) | -0.236 (-0.688, 0.217), p=0.308 | 0.052 (-0.103, 0.207), p=0.507 | -0.318 (-0.473, -0.163), p<0.001 | -0.091 (-0.241, 0.059), p=0.233 |
| Non-healthcare worker (vs nurse) | -0.008 (-0.418, 0.402), p=0.969 | -0.157 (-0.298, -0.015), p=0.031 | -0.793 (-0.944, -0.642), p<0.001 | -0.052 (-0.191, 0.087), p=0.467 |
| Years of clinical experience (years) | 0.026 (0.007, 0.046), p=0.009 | 0.004 (-0.003, 0.011), p=0.235 | 0.003 (-0.004, 0.010), p=0.387 | 0.007 (-0.000, 0.013), p=0.056 |
| Infection control committee participation | 0.068 (-0.973, 1.108), p=0.898 | 0.104 (-0.195, 0.403), p=0.495 | 0.217 (-0.059, 0.493), p=0.124 | 0.171 (-0.146, 0.488), p=0.292 |
| n | 1077 | 1077 | 1077 | 1077 |

Given that the binary classification of managerial positions may not fully reflect the occupational reality of a university hospital, we present the results of a sensitivity analysis using a model that excludes managerial positions. Values represent β coefficients and 95% confidence intervals. Nurses served as the reference category.

Table S6. Ordinal logistic regression (n = 1077)

| Independent variable | Risk perception OR (95% CI), p | Self-efficacy OR (95% CI), p | Sense of responsibility OR (95% CI), p | Attitude OR (95% CI), p |
| --- | --- | --- | --- | --- |
| Physician  (vs nurse) | 0.743 (0.537, 1.028), p=0.072 | 1.389 (0.989, 1.950), p=0.058 | 0.559 (0.396, 0.789), p=0.001 | 0.762 (0.538, 1.080), p=0.127 |
| Pharmacist  (vs nurse) | 1.606 (0.908, 2.841), p=0.105 | 1.246 (0.681, 2.280), p=0.476 | 0.979 (0.528, 1.815), p=0.948 | 1.559 (0.838, 2.900), p=0.164 |
| Other healthcare worker (vs nurse) | 0.812 (0.579, 1.140), p=0.229 | 1.100 (0.766, 1.580), p=0.606 | 0.482 (0.335, 0.695), p<0.001 | 0.778 (0.541, 1.119), p=0.174 |
| Non-healthcare worker (vs nurse) | 0.919 (0.663, 1.274), p=0.611 | 0.670 (0.469, 0.957), p=0.027 | 0.143 (0.099, 0.208), p<0.001 | 0.817 (0.574, 1.162), p=0.262 |
| Years of clinical experience (years) | 1.021 (1.004, 1.037), p=0.013 | 1.007 (0.989, 1.024), p=0.449 | 1.006 (0.988, 1.023), p=0.534 | 1.013 (0.995, 1.031), p=0.152 |
| Managerial position (leadership role) | 1.281 (0.905, 1.813), p=0.162 | 1.156 (0.806, 1.658), p=0.430 | 1.246 (0.862, 1.800), p=0.243 | 1.379 (0.949, 2.002), p=0.091 |
| Infection control committee participation | 1.600 (0.883, 2.899), p=0.122 | 1.464 (0.787, 2.725), p=0.228 | 1.833 (0.971, 3.460), p=0.062 | 2.065 (1.063, 4.011), p=0.032 |
| n | 1077 | 1077 | 1077 | 1077 |

To examine the validity of treating single-item dependent variables (Q4, Q5, and Q8) and risk perception score as continuous variables in the linear regression, ordinal logistic regression was conducted for each dependent variable. Values represent odds ratios (ORs) with 95% confidence intervals. Nurses served as the reference category.

**Table S7. Sensitivity analysis with the environmental score added as a covariate (n = 1,077)**

| **Independent variable** | **Risk perception β (95% CI), p** | **Self-efficacy β (95% CI), p** | **Sense of responsibility β (95% CI), p** | **Attitude β (95% CI), p** |
| --- | --- | --- | --- | --- |
| Physician (vs nurse) | -0.343 (-0.745, 0.059), 0.095 | 0.076 (-0.048, 0.201), 0.231 | -0.271 (-0.390, -0.152), <0.001 | -0.112 (-0.249, 0.026), 0.111 |
| Pharmacist (vs nurse) | 0.445 (-0.094, 0.984), 0.106 | -0.066 (-0.288, 0.157), 0.564 | -0.164 (-0.369, 0.041), 0.116 | 0.096 (-0.109, 0.302), 0.358 |
| Other healthcare worker (vs nurse) | -0.212 (-0.635, 0.211), 0.326 | 0.061 (-0.066, 0.187), 0.347 | -0.309 (-0.444, -0.173), <0.001 | -0.081 (-0.222, 0.060), 0.261 |
| Non-healthcare worker (vs nurse) | 0.272 (-0.161, 0.705), 0.218 | 0.030 (-0.083, 0.142), 0.605 | -0.648 (-0.779, -0.517), <0.001 | 0.054 (-0.086, 0.195), 0.450 |
| Years of clinical experience (years) | 0.020 (0.000, 0.040), 0.047 | 0.001 (-0.005, 0.006), 0.837 | 0.000 (-0.006, 0.006), 0.996 | 0.004 (-0.003, 0.011), 0.227 |
| Managerial position (leadership role) | 0.107 (-0.323, 0.536), 0.626 | 0.009 (-0.117, 0.135), 0.891 | 0.032 (-0.095, 0.158), 0.626 | 0.053 (-0.091, 0.198), 0.470 |
| Infection control committee participation | -0.051 (-1.052, 0.950), 0.921 | 0.040 (-0.199, 0.280), 0.742 | 0.161 (-0.100, 0.422), 0.227 | 0.123 (-0.172, 0.417), 0.415 |
| Environmental score (range 2–10) | 0.448 (0.325, 0.571), <0.001 | 0.304 (0.273, 0.335), <0.001 | 0.235 (0.200, 0.270), <0.001 | 0.168 (0.129, 0.206), <0.001 |

Values represent β coefficients, 95% confidence intervals (95% CI), and p-values. Nurses served as the reference category. The environmental score is the sum of the two environmental/organisational items (Q6 and Q7; range 2–10). HC3 robust standard errors were used.

**Table S8. Distribution of sex by occupational category (n = 1,077)**

| **Occupational category** | **Female, n (%)** | **Male, n (%)** | **Total** |
| --- | --- | --- | --- |
| Nurse | 475 (89.8) | 54 (10.2) | 529 |
| Physician | 63 (29.7) | 149 (70.3) | 212 |
| Pharmacist | 25 (61.0) | 16 (39.0) | 41 |
| Other healthcare worker | 73 (52.1) | 67 (47.9) | 140 |
| Non-healthcare worker | 131 (84.5) | 24 (15.5) | 155 |
| Overall | 767 (71.2) | 310 (28.8) | 1,077 |

**Table S9. Sensitivity analysis with sex (binary) added as a covariate (n = 1,077)**

| **Independent variable** | **Risk perception β (95% CI), p** | **Self-efficacy β (95% CI), p** | **Sense of responsibility β (95% CI), p** | **Attitude β (95% CI), p** |
| --- | --- | --- | --- | --- |
| Physician (vs nurse) | 0.018 (-0.444, 0.480), 0.939 | 0.103 (-0.061, 0.267), 0.219 | -0.152 (-0.301, -0.004), 0.044 | -0.025 (-0.178, 0.128), 0.747 |
| Pharmacist (vs nurse) | 0.856 (0.295, 1.418), 0.003 | 0.107 (-0.143, 0.358), 0.402 | 0.016 (-0.230, 0.262), 0.899 | 0.228 (0.021, 0.436), 0.032 |
| Other healthcare worker (vs nurse) | -0.012 (-0.485, 0.461), 0.960 | 0.036 (-0.130, 0.202), 0.672 | -0.256 (-0.419, -0.093), 0.002 | -0.038 (-0.193, 0.118), 0.633 |
| Non-healthcare worker (vs nurse) | 0.076 (-0.338, 0.490), 0.720 | -0.160 (-0.302, -0.019), 0.027 | -0.752 (-0.907, -0.598), <0.001 | -0.025 (-0.164, 0.115), 0.727 |
| Years of clinical experience (years) | 0.024 (0.003, 0.045), 0.025 | 0.003 (-0.004, 0.010), 0.407 | 0.002 (-0.005, 0.009), 0.530 | 0.006 (-0.002, 0.013), 0.129 |
| Managerial position (leadership role) | 0.194 (-0.251, 0.639), 0.393 | 0.062 (-0.083, 0.208), 0.402 | 0.074 (-0.064, 0.213), 0.292 | 0.086 (-0.068, 0.239), 0.273 |
| Infection control committee participation | 0.144 (-0.898, 1.185), 0.787 | 0.071 (-0.230, 0.373), 0.644 | 0.220 (-0.060, 0.500), 0.123 | 0.171 (-0.148, 0.491), 0.294 |
| Sex: male (vs female) | -0.447 (-0.827, -0.067), 0.022 | 0.059 (-0.074, 0.192), 0.382 | -0.101 (-0.229, 0.028), 0.125 | -0.083 (-0.204, 0.037), 0.175 |

Values represent β coefficients, 95% confidence intervals (95% CI), and p-values. Nurses served as the reference category for occupational categories, and female was the reference category for sex. HC3 robust standard errors were used.
